# Cross-cultural adaptation and validation of the Nursing Student Satisfaction Scale for use with Brazilian nursing students

**DOI:** 10.1590/1518-8345.1053.2776

**Published:** 2016-08-29

**Authors:** Carolina Domingues Hirsch, Edison Luiz Devos Barlem, Jamila Geri Tomaschewski Barlem, Graziele de Lima Dalmolin, Liliane Alves Pereira, Amanda Guimarães Ferreira

**Affiliations:** 2 Doctoral Student, Universidade Federal do Rio Grande, FURG, Rio Grande, RS, Brazil.; 3 Professor, Universidade Federal de Rio Grande, FURG, Rio Grande, RS, Brazil.; 4 Professor, Universidade Federal de Santa Maria, UFSM, Santa Maria, RS, Brazil.; 5 Master's Student, Universidade Federal do Rio Grande, FURG, Rio Grande, RS, Brazil.

**Keywords:** Nursing Student, Personal Satisfaction, Validation Studies

## Abstract

**Objective:**

to cross-culturally adapt and validate the Nursing Student Satisfaction Scale (NSSS) for use with nursing students in the Brazilian context.

**Method:**

this was a quantitative exploratory and descriptive study using a cross-sectional design conducted with 123 undergraduate nursing students studying at a public university in the south of Brazil. The cross-cultural adaptation was performed according to international guidelines. Validation for use in a Brazilian context was performed using factor analysis and Cronbach's alpha.

**Results:**

based on the expert committee assessment and pre-test, face and content validity were considered satisfactory. Factor analysis resulted in three constructs: curriculum and teaching; professional social interaction, and learning environment. The internal consistency of the instrument was satisfactory: the value of Cronbach's alpha coefficient was 0.93 for the instrument as a whole, and between 0.88 and 0.89 for the constructs.

**Conclusion:**

the Brazilian version of the Nursing Student Satisfaction Scale was shown to be reliable and validated for the evaluation of student satisfaction with undergraduate nursing programs, considering the aspects teaching activities, curriculum, professional social interaction, and learning environment.

## Introduction

Satisfaction can be understood as an individual's subjective perception of meeting his/her expectations in relation to various aspects of life^1^. The constant and dynamic state of change in modern society is driving a growing demand for qualified professionals with specialist knowledge and skills. 

One of the reflexes of this state of change is that the labor market has become more demanding. More rigorous qualification requirements mean that workers must constantly review, update and develop their knowledge, skills and competencies^2^. In addition, the educational landscape has undergone various changes in order to adapt to new global challenges. This reshaping of education aims to adapt teaching to the needs of society, resulting in structural changes in management models that lead to a constant need to rethink teaching strategies^3^. 

In this respect, student satisfaction can be viewed as an essential factor for motivating and involving students, thus enhancing the benefits of learning and, consequently, the professional competence of future professionals^4^. Student satisfaction involves the student's unique perception of the value of his/her educational experience during the degree program. Thus, satisfaction can be described as the level of harmony between what is demanded of the individual and what he/she expects or, in other words, as an individual's perception of whether his/her expectations are being met^3^.

University can promote significant changes to the life of students, while increasing demands and responsibilities may lead to feelings of anguish and fear caused by difficulties in adapting to a new social and cultural environment^5^. Academic demands and living in a new, and often hostile, environment, together with constant market pressure for increasingly efficient workers, can overburden students and lead to a perception of failure and lack of achievement of goals and expectations^2^. These limitations lead to demotivation and students begin to feel uninterested and dissatisfied. Satisfaction is the result of the complex and dynamic interaction between general living conditions, work relationships, the work process and an individual's perception of control over his/her living and working conditions^5^ ^).^ The fast pace of life and lack of concern with the personal needs of individuals are therefore intimately related to the perception of satisfaction.

However, the detection of factors that lead to student dissatisfaction can be complex due to their multifaceted nature: curriculum, teaching, professional social interaction, and learning environment^6^. The use of tools to measure student satisfaction and gain an insight into its different dimensions is therefore essential for evaluating educational programs with a view to improving the quality of education, adapting it to student needs and consequently reducing dropout rates.

The Nursing Student Satisfaction Scale (NSSS) was developed for use in quantitative studies to measure nursing student satisfaction with nursing programs^6^. The instrument was validated in the United States with a sample of 303 nursing students and consists of questions that address curriculum and teaching, professional social interaction, and the learning environment. 

In Brazil, few studies exist on student satisfaction in the specific context of nursing. It is therefore essential to develop instruments that help us to understand the factors that affect student satisfaction with nursing programs in order to improve the quality of education and adapt programs to students' needs, thus enhancing student satisfaction and reducing dropout rates.

The justification for this study is thus based on the need to analyze the factors that caused the demotivation during the degree program, thereby making it possible to improve the processes for meeting needs with a view to increasing the satisfaction of Brazilian nursing academics, exploring the dimensions of teaching, curriculum, professional social interaction and the learning environment. This study therefore aims to validate and cross-culturally adapt the Nursing Student Satisfaction Scale (NSSS) to the specific context of Brazilian nursing students.

## Method

This was a quantitative exploratory and descriptive study using a cross-sectional design. The cross-cultural adaptation and validation of the NSSS for use in the Brazilian context was performed drawing on concepts from relevant international scientific literature^7^. The validation process involved the translation and retranslation of the items of the original English version of the instrument into Brazilian Portuguese to test its face and content validity, while the description of the psychometric properties related to its construct validity and reliability was measured using factor analysis and Cronbach's alpha.

Authorization was obtained from the original developer of the NSSS to culturally adapt the instrument. All ethical concerns were considered and addressed in accordance the National Health Council Resolution 466/12 and the study was approved by the local Research Ethics Committee.

### The original instrument

The original version of the NSSS in English is composed of 30 questions and aims to measure student satisfaction with educational programs. Thirty questions in three subscales were validated by administering the NSSS among a sample of 303 nursing students: 14 items concerning *curriculum and teaching*; nine items concerning *professional social interaction*, and six items related to *the learning environment*. The scale also contains a specific item that assesses student satisfaction. The NSSS uses a 6-point Likert scale scored from 1 (not at all satisfied), to 6 (very satisfied).

### Cross-cultural adaptation: face and content validity

According to international cross-cultural adaptation guidelines, cross-cultural adaptation was performed in six stages in order to obtain semantic, linguistic, experiential and conceptual equivalence: initial translation; synthesis; back translation; expert committee; pretesting; and review of the adaptation process by the researchers^7^.

For the first stage, the instrument was translated from English to Portuguese by two bilingual translators. One of the translators was informed of the aims and topic of the collection instrument, while the other had no knowledge of the aims and topic. The two translations were then synthesized into a single version (synthesized version). Subsequently, the synthesized version was back translated into English by two other translators. Neither of the translators was informed of the content and aims of the instrument in order to avoid mistaken meanings. 

The back translation was then evaluated by an expert committee made up of four professors who hold doctorates and have extensive experience in nursing research. The committee evaluated the semantic, cultural, linguistic, and conceptual equivalence and the face validity of the scale and approved it for pretesting, developing a pre-final version of the instrument. This validated version was administered among a sample of 30 nursing students from masters and PhD nursing programs offered by a public university in the south of Brazil. 

The aim of the pre-test was to confirm whether the items contained in the scale represented the intended content. The questionnaires were administered individually so that each participant could highlight the difficulties or easiness encountered in completing the instrument and suggest changes to the questions if necessary^7^. Finally, the cross-cultural adaption process was reviewed, whereby the researchers made the necessary changes to the scale in order to facilitate its understanding by the selected sample.

After following these cross-cultural adaptation procedures, the final Brazilian version of the NSSS was approved for use in the Brazilian context. 

### Location and study participants

The final version of the scale was administered in a federal university in the south of Brazil that offers free education and whose purpose is to promote higher education, research and extension. Convenience sampling was used to select the study sample, whereby study participants were selected according to their presence and availability in the location at the time of data collection^8^. A specific formula was used to determine the minimum sample size needed for statistical analysis^9^. Based on a previously known population of 187 nursing students, the formula resulted in a minimum study sample of 123 study participants.

### Data collection

The scale was administered collectively during the normal lecture period with the authorization of the nursing faculty. After the procedures related to the ethical aspects of the study were undertaken, the scales were placed in a brown envelope and handed directly to the participants, who completed the questionnaire anonymously.

### Construct validation of the scale

After administering the questionnaire, statistical tests were performed using the SPSS (Statistical Package for Social Sciences) version 22.0 to measure the clarity and reliability of the Brazilian version. Two tests were performed to measure construct validity: factor analysis, and Cronbach's alpha^10^. 

With respect to factor analysis, the data was summarized by identifying factors common to each question and grouping the questions into categories (constructs) based on the average of the answers. Cronbach's alpha was used to estimate the reliability of the instrument by assessing the characteristics of each group of questions to determine whether the questions contained in the scale consistently measured the phenomenon in question ^(^ ^10^.

Principal component analysis was used as the extraction method. Varimax orthogonal rotation was used to determine the relevance of the variables to the identified components. The formation of the factors was based on two criteria: the degree of relationship between the variables, determined by the factor loadings (>.400); and degree of subjectivity^10^. 

## Results

With respect to face validity, there was a consensus among the expert committee that all items were relevant and demonstrated semantic, cultural, linguistic and conceptual equivalence. All items and question formulation were understandable and therefore changes to the questions were limited to how they were written.

The Likert scale was reduced from six to five points in order to create a midpoint response for respondents to be able to express neutrality between satisfaction and dissatisfaction. The six-point scale, initially comprising of 1 ("Not at all satisfied"), 2 "Not very satisfied", 3 ("Slightly dissatisfied", 4 ("Relatively satisfied"), 5 ("Satisfied"), and 6 ("Very satisfied"), was replaced by 1 ("Not at all satisfied"), 2 ("slightly satisfied"), 3 ("neither satisfied nor dissatisfied"), 4 ("satisfied"), and 5 ("totally satisfied"). 

It was also suggested that question 16 - "*Eu acredito que o currículo de enfermagem me preparou para fazer o exame NCLEX-RN*" (Program prepared me to take the NCLEX-RN) - should be reformulated to "*Acredito que o currículo de enfermagem me preparou para realizar o exame ENADE*" (Program prepared me to take the *ENADE* exam) to culturally adapt it to the context of Brazilian students.

With respect to content validity, the pre-test carried out with 30 nursing students from masters and/or PhD nursing programs showed that the items represented the content analyzed, requiring only small modifications to questions 1, 2, 5, 6, 9, 17, and 28. The time needed to fill out the questionnaire varied from between 10 and 20 minutes.

With respect to question 1 - "*O currículo de enfermagem está aprimorando minha capacidade de resolver problemas ao cuidar dos pacientes*" (the program is enhancing my problem-solving skills when it comes to patient care) - it was suggested that the term "*aprimorando*" should be replaced by "*desenvolvendo*" (developing) given that the training process is still underway. Question 1 was therefore formulated as follows: "*O currículo de enfermagem está desenvolvendo minha capacidade de resolver problemas ao cuidar dos pacientes*" (the nursing curriculum is developing my problem-solving capacity when it comes to patient care).

With respect to question 2 - "*O corpo docente de enfermagem é bem qualificado em sua* área" (the nursing faculty are well qualified in their field) - it was suggested that "*de atuação*" (of practice) should be added, resulting in the following formulation: "*O corpo docente de enfermagem é bem qualificado em sua* área *de atuação*" (The nursing faculty are well qualified in their field of practice). Question 5 - "*Os docentes de enfermagem estão sendo modelos positivos de enfermagem profissional*" (the faculty are positive role models of professional nursing) was reformulated semantically as follows: "*Os docentes de enfermagem estão sendo modelos positivos de profissionais*" (the nursing faculty are professional role models). With regard to question 6 - "*Eu sou respeitado pela equipe de enfermagem dentro do ambiente clínico*" (I am respected by the nursing staff in the clinical setting) - it was suggested that the term "*ambiente clínico*" should be replaced by "*ambiente das práticas clínicas*" (clinical practices environment) as follows: "*Eu sou respeitado pela equipe de enfermagem dentro do ambiente das práticas clínicas*" (I am respected by the nursing staff in the clinical practices setting). With respect to question 9 - "*Os membros do corpo docente de enfermagem atuam de forma colaborativa entre si no processo de ensino*" (Nursing faculty members collaboratively work with each other in the teaching process) - it was suggested that the terms "*membros do corpo*" and "*de enfermagem*" should be removed. The question was therefore reformulated as follows: "*Os docentes atuam de forma colaborativa entre si no processo de ensino*" (The faculty collaboratively work with each other in the teaching process). 

With regard to question 17 - "*Os docentes de enfermagem explicam conceitos essenciais com eficácia*" (The faculty effectively explain essential concepts) - it was suggested that the term "*com eficácia*" should be replaced by "*para o exercício da profissão de forma efetiva*" (of nursing practice effectively) as follows: "*Os docentes de enfermagem explicam conceitos essenciais para o exercício da profissão de forma efetiva*" (The faculty effectively explain essential concepts of nursing practice). With respect to question 28 - "*Os docentes de enfermagem estão tendo boas expectativas com o meu desempenho*" (The faculty have good expectations of my performance) - the term "*Percebo que*" was added to the beginning of the question as follows: "*Percebo que os docentes de enfermagem estão tendo boas expectativas com o meu desempenho*" (I realize that the faculty have good expectations of my performance).

After the expert committee's assessment of the questionnaire and application of the pre-test, the culturally adapted instrument was administered among the sample to measure construct validity and evaluate its psychometric properties. Of the sample of 123 students, 112 (91.05%) were female and 11 (8.9%) were male. The age of the sample varied between 18 and 50 years and average was 25.36 years. The majority of students were single (79.7%) and did not have children (81.3%). Over half of the students practiced extracurricular activities (59.4%), of which 49.6% received some form of scholarship. The majority of students did not work (77.2%), while 13.8% stated that they worked in the health field. 

A significant proportion of students were in the first semester of the course (16.3%) and the majority (72.6%) confirmed that nursing was their first choice of course. The majority of students stated that they made an informed decision in choosing the course (70.07%), while 61.8% stated that they had never thought of dropping out of the course.

With respect to construct validity, the instrument's 30 questions were subjected to exploratory factor analysis (of blocks) to determine discriminant validity. The first grouping resulted in the formation of five constructs, which hindered categorization within the proposed framework. Questions that had low correlations within their blocks were therefore gradually excluded in order to group the questions based on a factor loading cut-off point of > 0.400 for the formation of each construct. This procedure resulted in the exclusion of seven questions from the instrument.

The three dimensions of the instrument account for 54.20% of the variation between the original questions, which represents an adequate degree of data synthesis, thus facilitating data handling and interpretation.

The reliability of the instrument's three constructs was tested by calculating Cronbach's alpha. The value of Cronbach's alpha coefficient for the instrument as a whole was 0.93, considering that the coefficients of the three constructs ranged between 0.88 and 0.89, which is considered high for an exploratory study, thus confirming the reliability of the scale for the selected sample. Table 1 shows the factor loading of each construct according to the formation of factors, explained variance and Cronbach's alpha coefficient values. 

**Table 1:** Exploratory factor analysis (Varimax rotation). State of Rio Grande do Sul, Brazil, 2015

Indicators		Block	F1	F2	F3
Professional Social Interaction					
	S05 *Os docentes de enfermagem estão sendo modelos positivos de profissionais* (the nursing faculty are professional role models)	0.670	.776		
	S10 *Eu me sinto tranquilo ao fazer questionamentos ao corpo docente de enfermagem* (I feel comfortable asking questions of the faculty)	0.646	.766		
	S03 *Eu sou respeitado pelo corpo docente* (I am respected by the faculty)	0.596	.738		
	S25 *Os docentes de enfermagem são justos/imparciais ao avaliar o meu aprendizado* (The faculty is fair/unbiased in their assessment of my learning)	0.600	.716		
	S18 *Eu tenho interações profissionais positivas com os docentes de enfermagem* (I have positive professional interactions with my faculty)	0.594	.713		
	S21 *Os professores de enfermagem fazem um esforço para deixar as matérias interessantes* (The nursing faculty make an effort to make their topics interesting)	0.476	.666		
	S17 *Os docentes de enfermagem explicam conceitos essenciais para o exercício da profissão de forma efetiva* (The faculty effectively explain essential concepts of nursing practice)	0.607	.630		
	S02 *O corpo docente de enfermagem é bem qualificado em sua área de atuação* (The nursing faculty are well qualified in their field of practice)	0.469	.593		
	S09 *Os docentes atuam de forma colaborativa entre si no processo de ensino* (The faculty collaboratively work with each other in the teaching process)	0.449	.570		
Curriculum and Teaching					
	S01 *O currículo de enfermagem está desenvolvendo minha capacidade de resolver problemas ao cuidar dos pacientes* (the nursing curriculum is developing my problem-solving capacity when it comes to patient care)	0.645		.767	
	S20 *O currículo de enfermagem está me preparando para eu me tornar um enfermeiro competente* (The nursing program is preparing me to become a competent nurse)	0.702		.726	
	S08 *O currículo de enfermagem está me capacitando para utilizar o processo de enfermagem na prática clínica* (The nursing program is preparing me to use the nursing process in clinical practice)	0.618		.700	
	S04 *O currículo de enfermagem está me ajudando a aprimorar minhas habilidades comunicativas* (The nursing program is helping me to improve my communication skills)	0.510		.679	
	S24 *O currículo de enfermagem* é relevante para a atual *prática de enfermagem* (The nursing program is relevant to current nursing practice)	0.581		.653	
	S29 *O currículo de enfermagem progride de forma lógica de conceitos simples a complexos* (The nursing program progresses logically from simple to complex concepts)	0.533		.642	
	S12 *Eu me sinto confiante na minha habilidade de atuar em ambientes clínicos em razão do O currículo de enfermagem* (I feel confident about my ability to act in clinical settings due to the program)	0.506		.631	
Learning environment					
	S15 *Os equipamentos no laboratório de enfermagem estão em bom estado de conservação* (The equipment in the nursing lab is in good repair)	0.720			.823
	S11 *Os equipamentos do laboratório de enfermagem estão atualizados* (The equipment in the nursing lab is up to date)	0.711			.800
	S19 *Há equipamentos suficientes no laboratório de enfermagem para a minha aprendizagem* (Laboratory resources are adequate for my learning needs)	0.693			.785
	S23 *O laboratório de enfermagem tem espaço suficiente para a minha aprendizagem* (The nursing lab has ample space for my learning needs)	0.632			.748
	S26 *Os recursos da biblioteca são adequados para a aprendizagem* (Library resources are adequate for my learning needs)	0.544			.665
	S13 *Os docentes usam tecnologia de forma eficaz para melhorar meu aprendizado* (The faculty effectively use technology to enhance my learning)	0.596			.619

% explained variance - after rotation (59.546%) Cronbach's alpha coefficient (instrument 0.934).

KMO measure of sampling adequacy (KMO = 0.880).

Bartlett's test: chi-square = 1441.960.

**Figure 1:**
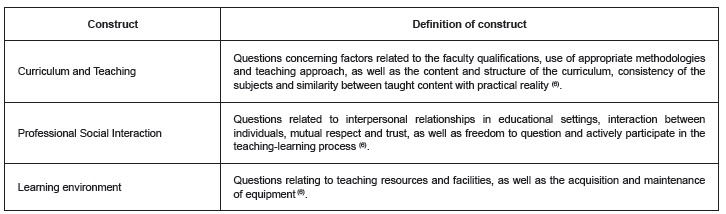
Definition of the constructs that directly affect student satisfaction developed by factor analysis - the State of Rio Grande do Sul 2015.

The final version of the instrument was therefore composed of three constructs made up of 22 items: curriculum and teaching; professional social interaction, and learning environment, as shown in Figure 1. 

## Discussion

The inclusion of student satisfaction as a course evaluation indicator can provide important information and insights into students' expectations and perceptions of the educational experience, which can be used to promote teaching and program development and enhancement ^(^ ^11^. Therefore, the validated Portuguese version of the NSSS is an important tool for identifying the factors that determine nursing student satisfaction in the Brazilian context. 

The findings show that the three constructs of the Brazilian version have slight differences to those of the original version in terms of structure and conceptual definition ^(^ ^6^. The instrument provides a theory-based approach to measuring student satisfaction by revealing the association between its dimensions and student satisfaction with the course. It is also important to highlight that this version of the NSSS is the first to be made available in Brazil and as such other versions that address the specific context of Brazilian students do not exist in the literature.

With respect to the structure of the instrument, the expert committee suggested that the Likert scale be reduced from six to five points to create a midpoint response for respondents to be able to express neutrality between satisfaction and dissatisfaction, which is not possible in six-point scales. 

Given the importance of the expert committee assessment, it is vital to select professionals who have adequate knowledge to enable them to examine not only the semantic content of the questions, but also other aspects of the instrument such as structure and layout^12^.

The original NSSS was composed of three constructs ^-^ curriculum and teaching, professional social interaction, and learning environment^6^ ^) -^ containing 30 validated questions, as opposed to 22 validated questions in the Brazilian version. The differences in the results of the administration of the NSSS in different settings and cultures show that students' perception of the factors that determine satisfaction with the course varies in different contexts^2^.

The characteristics of the validation process differ depending on the purpose of the validation, which can be classified in two ways: validation of the instrument for use in a new context; and validation for cultural studies involving different versions of the same instrument ^12^. 

The first construct of the Brazilian version of the NSSS, *professional social interaction*, was made up of items that were included in the first and second subscales of the original NSSS ^6^, *curriculum and teaching* and *professional social interaction*. The relational problems evident in the training environment affect students' perceptions of satisfaction with the course and are reflected in difficulties in adapting to teaching methods and practices ^13^. The items in this construct group address the social interaction among students and faculty and how this affects the teaching-learning process. This construct was shown to be relevant given that negative student-faculty interactions lead to dissatisfaction and make students think about dropping out of the course^3^.

The construct *curriculum and teaching* was shown to be directly related to the subscale *curriculum and teaching* of the original NSSS ^6^, comprising of questions relating to qualifications and preparedness for teaching, use of appropriate methodologies and teaching approach, as well as the content and structure of the curriculum, consistency of the subjects and similarity between taught content with practical reality. However, in the original NSSS, the *curriculum and teaching* subscale also includes items that address social interaction among students and faculty and how this affects teaching-learning, whereas in the Brazilian version this construct is restricted to questions that address only curriculum and teaching. These other factors were grouped into the first construct or excluded from the instrument. Therefore, this dimension was consistent with the literature since issues relating to curriculum are seen by students as important factors affecting general satisfaction with the educational programs^6^ ^-^ ^14^.

With respect to the final construct, *learning environment*, the validated items were the same as those in the original version, except for two items: one excluded because it had low factor loading; and an item that was added because it was conceptually related to the construct. The items of this construct address learning environment resources and facilities and the modernization of technological learning methods that enhance information sharing^14^. This construct was also consistent with the literature, since adequate resources and facilities support students and enhance the theoretical and practical knowledge acquired throughout educational programs^15^.

The results obtained with respect to reliability were very satisfactory when compared to the validation of the original version^6^, thus guaranteeing the reliability of the validated instrument for future studies. The value of Cronbach's alpha coefficient for the Brazilian version of the NSSS was 0.93, while the values for the three constructs ranged between 0.88 and 0.89. These values are similar to those of the original NSSS: Cronbach's alpha coefficients of internal consistency of the 30 items were 0.93 for the overall scale and between 0.85 and 0.88 for the three constructs^6^.

## Conclusion

The findings show that the Brazilian version of the Nursing Students Satisfaction Scale is a sound approach for measuring student satisfaction with educational programs and understanding the factors that may demotivate students during the teaching-learning process. It was possible to identify three factors that affect student satisfaction with a nursing program within the Brazilian context: Professional social interaction; Curriculum and teaching, and learning environment.

The Portuguese version of the NSSS is an important tool and the validation process provided important insights into the factors that potentially affect the satisfaction of students with the course and the attractiveness of universities to students in a Brazilian context. 

The primary limitations of this study were the lack of NSSSs adapted and validated for use in other countries, which made further comparisons impossible, and the fact that the study was limited to a specific group of students in a public university in the south of Brazil. 

Finally, it is recommended that this version of the NSSS be evaluated in other locations in Brazil to determine whether there are significant differences in the factors that affect student satisfaction with other nursing programs.
